# Adjusted CT Image-Based Radiomic Features Combined with Immune Genomic Expression Achieve Accurate Prognostic Classification and Identification of Therapeutic Targets in Stage III Colorectal Cancer

**DOI:** 10.3390/cancers14081895

**Published:** 2022-04-08

**Authors:** Yi-Ching Huang, Yi-Shan Tsai, Chung-I Li, Ren-Hao Chan, Yu-Min Yeh, Po-Chuan Chen, Meng-Ru Shen, Peng-Chan Lin

**Affiliations:** 1 Department of Internal Medicine, National Cheng Kung University Hospital, College of Medicine, National Cheng Kung University, Tainan 704, Taiwan; n102114@mail.hosp.ncku.edu.tw; 2 Department of Oncology, National Cheng Kung University Hospital, College of Medicine, National Cheng Kung University, Tainan 704, Taiwan; s98031083@mail.ncku.edu.tw; 3 Department of Medical Imaging, National Cheng Kung University Hospital, College of Medicine, National Cheng Kung University, Tainan 704, Taiwan; n506356@mail.hosp.ncku.edu.tw; 4 Department of Statistics, National Cheng Kung University, Tainan 704, Taiwan; cili@mail.ncku.edu.tw; 5 Department of Surgery, National Cheng Kung University Hospital, College of Medicine, National Cheng Kung University, Tainan 704, Taiwan; n803421@mail.hosp.ncku.edu.tw (R.-H.C.); n040581@mail.hosp.ncku.edu.tw (P.-C.C.); 6 Institute of Clinical Medicine, National Cheng Kung University Hospital, College of Medicine, National Cheng Kung University, Tainan 704, Taiwan; mrshen@mail.ncku.edu.tw; 7 Department of Obstetrics and Gynecology, National Cheng Kung University Hospital, College of Medicine, National Cheng Kung University, Tainan 704, Taiwan; 8 Department of Pharmacology, National Cheng Kung University Hospital, College of Medicine, National Cheng Kung University, Tainan 704, Taiwan; 9 Department of Genomic Medicine, National Cheng Kung University Hospital, College of Medicine, National Cheng Kung University, Tainan 704, Taiwan; 10 Institute of Medical Informatics, College of Electrical Engineering and Computer Science, National Cheng Kung University, Tainan 704, Taiwan

**Keywords:** covariate adjustment, adjusted radiomics, immune genomic expressions, cancer recurrence, therapeutic targets

## Abstract

**Simple Summary:**

Using the covariate-adjusted tensor classification in the high-dimension (CATCH) model, we integrated adjusted radiomics-based CT images into RNA immune genomic expression data to achieve the accurate classification of recurrent CRC. The correlation between radiomic features and immune gene expression identifies potential therapeutic targets in CRC. We provide individualized cancer therapeutic strategies based on adjusted radiomic features in recurrent stage III CRC.

**Abstract:**

To evaluate whether adjusted computed tomography (CT) scan image-based radiomics combined with immune genomic expression can achieve accurate stratification of cancer recurrence and identify potential therapeutic targets in stage III colorectal cancer (CRC), this cohort study enrolled 71 patients with postoperative stage III CRC. Based on preoperative CT scans, radiomic features were extracted and selected to build pixel image data using covariate-adjusted tensor classification in the high-dimension (CATCH) model. The differentially expressed RNA genes, as radiomic covariates, were identified by cancer recurrence. Predictive models were built using the pixel image and immune genomic expression factors, and the area under the curve (AUC) and F1 score were used to evaluate their performance. Significantly adjusted radiomic features were selected to predict recurrence. The association between the significantly adjusted radiomic features and immune gene expression was also investigated. Overall, 1037 radiomic features were converted into 33 × 32-pixel image data. Thirty differentially expressed genes were identified. We performed 100 iterations of 3-fold cross-validation to evaluate the performance of the CATCH model, which showed a high sensitivity of 0.66 and an F1 score of 0.69. The area under the curve (AUC) was 0.56. Overall, ten adjusted radiomic features were significantly associated with cancer recurrence in the CATCH model. All of these methods are texture-associated radiomics. Compared with non-adjusted radiomics, 7 out of 10 adjusted radiomic features influenced recurrence-free survival. The adjusted radiomic features were positively associated with *PECAM1*, *PRDM1*, *AIF1*, *IL10*, *ISG20*, and *TLR8* expression. We provide individualized cancer therapeutic strategies based on adjusted radiomic features in recurrent stage III CRC. Adjusted CT scan image-based radiomics with immune genomic expression covariates using the CATCH model can efficiently predict cancer recurrence. The correlation between adjusted radiomic features and immune genomic expression can provide biological relevance and individualized therapeutic targets.

## 1. Introduction

Colorectal cancer (CRC) is a common malignancy that results in significant morbidity and mortality. Abdominal computed tomography (CT) scans of the primary tumor are valuable in planning surgery for patients with stage II–III CRC because they can demonstrate the regional extension of the tumor, adenopathy, and distant metastases. However, the role of non-invasive CT scan imaging with respect to the tumor microenvironment (TME) remains unclear. CT scan-based radiomics can be used to extract high-dimensional imaging radiomic features. Radiomics has shown great potential as an excellent method for predicting recurrence in various types of cancer [1]. Some studies have applied CT-based radiomic features for the clinical evaluation, such as staging, recurrence, or lymph node metastasis prediction of patients with CRC [2,3,4,5]. A previous study calculated the clinical factors and radiomic scores to predict the recurrence risk in patients with stage II CRC [1].

In contrast to traditional imaging features, radiomics has been proposed to reveal the characteristics of the TME and genetic features. The TME is heterogeneous and consists of tumor, stromal, and immune cells. Tumor cell types and their environments affect cancer growth and metastasis. Almost every immune cell-containing cancer can be imaged using computed tomography (CT). Tumor image-derived texture features are associated with the cancer immune cell infiltration status [6]. Several potential molecular and TME-based immune predictors of recurrence risk and immunotherapy response have been recently investigated [7]. RNA sequencing methods can process a mixture of immune cells, averaging out the underlying differences in immune cell type-specific transcriptomes. RNA gene expressions of multiple tumor tissue immune cells that affect radiomic parameters are also present in imaging and radiomic features.

However, few studies have considered the covariates of texture radiomic features and integrated TME-based immune RNA expression into radiomic signatures to predict colon cancer prognosis. Another major challenge in studies of cancer recurrence prediction by radiomic features is that they are often composed of a relatively small number of patient samples and a large number of radiomic features. These types of data present a problem of high dimensionality. A good strategy is to reduce the number of dimensions using feature selection. This study aimed to evaluate the usefulness of adjusted CT scan image-based radiomics combined with immune genomic expression for the accurate stratification of cancer recurrence and identification of potential therapeutic targets in stage II–III CRC. We hypothesized that RNA gene expression affects radiomic features through the tumor microenvironment. Towards this goal, we used the covariate-adjusted tensor classification in the high-dimensional (CATCH) algorithm.

The main aim of the CATCH algorithm is to construct an interpretable discriminant analysis model for achieving variable selection and prediction consistency, even when the number of interesting variables is much larger than the sample size. Taking advantage of the CATCH algorithm, RNA expression was merged as a covariate into the radiomics-based model, offering a more comprehensive prediction of tumor recurrence. Based on the successful application of radiomics analyses in precision oncology, we constructed a relationship between RNA expression and radiomics to provide an accessible method for identifying potential therapeutic targets.

## 2. Materials and Methods

### 2.1. Patient Selection

This cohort study initially enrolled 99 patients with stage II–III CRC who underwent surgery, followed by adjuvant chemotherapy with leucovorin (folinic acid), fluorouracil, and oxaliplatin (FOLFOX), at the National Cheng Kung University Hospital (NCKUH) between January 2015 and January 2017. Eligible cancer patients were aged >= 20 years, as well as having an Eastern Cooperative Oncology Group performance status (ECOG PS) of 0–1, and adequate organ function. Follow-up continued through to January 2019. Primary tumor tissues were collected from all subjects for RNA immune response genes and CT scans for research purposes. To study the impact of immune response-associated gene expression and radiomics on recurrence, tumor samples from 99 high-risk patients were collected. After quality control, samples from 71 patients were retained for further analysis. Among the 71 patients, 21 patients (29.5%) had tumor recurrence. The tumor recurrence was defined as any tumor-related lesion, including local/regional or distant metastasis, first detected after the curative operation.

This study was approved by the Institutional Review Board of NCKUH (A-ER-103-395, A-ER-104-153, and B-ER-109-154) and was conducted according to the tenets of the Helsinki Declaration. All participants provided written informed consent.

### 2.2. Image Acquisition and Imaging Texture Analysis

Seventy-one consecutive CRC patients underwent pre-treatment abdominal/pelvic CT scans with and without intravenous (IV) contrast enhancement with an injected volume of 80 mL (iohexol, 350 mgI/ML or iopamide, 370 mgI/mL) scanned at the portal-to-delayed phase. All patients were studied, including 28 patients using 16-row CT scans (Sensation 16, Siemens Medical Solution, and BrightSpeed Series CT systems, General Electric (GE) Healthcare, Milwaukee, WI, USA), 15 patients using 64-row CT scans (Optima CT660 and LightSpeed, GE, and Sensation 64 and SOMATOM Definition AS, Siemens), and 28 patients using 128-row C T scans (SOMATOM Definition FLASH, Siemens Healthineers, Forchheim, Germany). For tumor segmentation, all portal venous phase CT images in the Digital Imaging and Communications in Medicine (DICOM) format were retrieved from the picture archiving and communication system (PACS) at NCKUH. The volume of interest (VOI) of the target tumors in serial slices was manually labeled by two senior board-certified radiologists using a self-developed image-labeling tool running on “INFINITE” PACS 3.0. The DICOM images were saved as the Neuroimaging Informatics Technology Initiative file type, and the mask using polygonal annotation was saved as the file format of nearly raw raster data. PyRadiomics uses SimpleITK for image loading and handling. Features were calculated by several built-in filters. These included wavelet and Laplacian of Gaussian (LoG) with sigma filters. In total, 1037 radiomic features were selected, and are shown in Table S1. To test the reproducibility of the image features, we randomly selected 30 patients with tumor labeling performed by two radiologists who were both blinded to the clinicopathological and outcome details. We assessed the radiologist’s reproducibility of double segmentation in CT scans. The median value of intra-class-correlation coefficients (ICCs) is 0.93. For the accuracy of the tumor labeling, we selected the radiomic features based on the two radiologists’ consensus. Quantitative imaging features were subsequently extracted from previously identified VOIs. The definitions of the radiomic features were derived from the PyRadiomics library (version 3.0). The PyRadiomics community maintains copyright for the definitions mentioned (http://github.com/radiomics/pyradiomics, accessed on 22 December 2021).

### 2.3. Radiomics Workflow and Feature Extraction

Radiomics is a feature extracted from diagnostic medical images using advanced feature-analysis algorithms. After tumor segmentation, we measured 1037 radiomic features, as summarized in Table S2. The following radiomic features were included for feature computation: (1) first-order statistics (19.1%), (2) gray level co-occurrence matrix (25.5%), (3) gray level dependence matrix (14.9%), (4) gray level run length matrix (17.0%), (5) gray level size zone matrix (17.0%), (6) neighboring gray tone difference matrix (5.3%), and (7) shape (1.4%). The spectrum and distribution of radiomic features are listed in Table S2. Based on the study by Khalifa et al. (2020) [8], 1037 radiomic feature data were converted into 33×32-pixel image data, representing a transformed image, using a feature extraction algorithm.

### 2.4. Tumor Microenvironment-Based RNA Immune Response Gene Sequencing

Cancer tissues with immune response gene expression profile data were obtained from 71 CRC patients. RNA was extracted from formalin-fixed paraffin-embedded tissue using the RecoverAll Total Nucleic Acid Isolation Kit (Thermo Fisher Scientific). The 398 RNA immune response genes were constructed into libraries using the Ion AmpliSeq Kit for Chef DL8 with the Ion Chef System. Raw gene expression data were preprocessed using Torrent Suite, followed by further normalization.

### 2.5. Statistical Analysis for Clinical Data

The chi-square test and Fisher’s exact test were used to assess the differences between the tumor recurrence and non-tumor recurrence groups. The tumor recurrence was defined as any tumor-related lesion, including local/regional or distant metastasis, first detected after the curative operation. Kaplan–Meier curves were used to evaluate recurrence-free survival (RFS), which was defined as the time between surgery and cancer recurrence. A *p*-value < 0.05 was considered statistically significant.

### 2.6. CATCH Model

**Figure 1:**
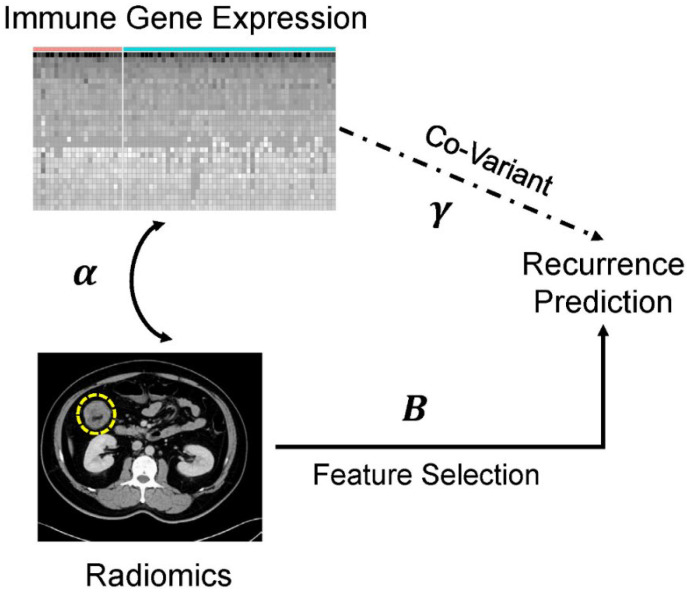
CATCH model for predicting risk of recurrence. The CATCH model can be used to reduce the influence of RNA gene expression covariates in radiomics data. *γ* represents the discriminative coefficients for the impact of immune gene expression on recurrence. α represents the radiomics parameters indicative of the indirect effect of immune gene expression on cancer recurrence. B represents the direct effect of radiomic features on recurrence in the tensor discriminant analysis.

To integrate radiomic feature information and immune response-associated gene expression profile data, the CATCH model proposed by Pan et al. [9] was used to predict the recurrence in CRC patients. The CATCH model is based on Bayes’ rule and is defined as follows:

$$\hat{Y}=arg\;\underset{k=1,2}{\max}\mathrm{Pr}\left\{a_{k}+\gamma_{k}^{T}U+\langle B_{k},X-\alpha_{\left(M+1\right)}\bar{\times }U\rangle \right\}$$

where $\hat{Y}$ is the predictor for categorical variable Y with two levels (1 for recurrent patients and 2 for non-recurrent patients), *X* represents the 33 × 32-pixel image data, and *U* represents the immune response-associated gene expression profile data. The parameters $\left\{\gamma_{k},\alpha ,B_{k}\right\}$ in Equation [9] are useful for clinical judgment. The coefficient $\gamma_{k}$ represents the direct effect of the immune response-associated gene expression profile (*U*) on recurrence (*Y*). The coefficient α represents the relationship between the radiomic features (*X*) and the immune response-associated gene expression profile. The coefficient $B_{k}$ represents the effect of *X* after adjusting for the covariate *U*, and $X^{adj}=X-\alpha_{\left(M+1\right)}\bar{\times }U$ represents the adjusted radiomic features. The diagram of the relationships between the coefficients $\left\{\gamma_{k},\alpha ,B_{k}\right\}$, which are critical and can guide the clinician to interpret the results obtained from the CATCH model, is shown in Figure 1. We described the method for using the coefficient $B_{k}$ and adjusted radiomic features $X^{adj}$ to build an individualized cancer therapeutic strategy.

## 3. Results

### 3.1. Patient Characteristics

To predict cancer recurrence based on radiomic features, we adjusted the covariates of the TME-based RNA gene expression. First, differentially expressed genes (DEGs) that affected cancer recurrence ($\gamma_{k}$) were identified [10]. Second, we demonstrated that the performance of the CATCH model based on radiomic features and RNA expression ($B_{k}$) affects clinical outcomes. Third, a correlation between radiomic features and RNA gene expression (α) was established to identify a potential therapeutic target (Figure 1). The results show that this model can successfully predict cancer recurrence. The baseline characteristics of the patients are presented in Table S3. In total, 49.3% were men, and the median patient age was 58 years. The distribution of gender was almost the same between patients with and without cancer recurrence (Table S3). Overall, 71.8% of CRC patients were aged <65 years. The primary tumors were most commonly located in the left colon (78.9%). Most patients had a high tumor invasive stage (T3–T4) (87.3%) and low tumor nodal stage (N0–N1) (70.4%). There was no significant difference in clinical characteristics between patients with and without cancer recurrence (Table S3). In the genetic features of colorectal cancers, there was no significant difference in mismatch repair (MMR), KRAS, and BRAF status between recurrence and no-recurrence groups. No prognostic risk factors were identified in clinicopathological and genetic features. In our dataset, the percentage of local/regional and distal recurrence was 23.8% and 76.2%, respectively. Among the 21 patients with tumor recurrence, one (4.8%) patient had local recurrence, and four (19.0%) patients had regional recurrences. Sixteen (76.2%) patients had distant metastases. Seven (33.3%) patients had lung-only metastasis. Three (14.3%) patients had liver-only metastasis (Table S4).

### 3.2. Identification of Genes Influencing Recurrence and Performance of the CATCH Model

To determine the correlation between RNA immune gene expression and clinical outcome, 30 significant DEGs were selected from 398 RNA genes. A differential gene expression analysis was performed using the DESeq2 R package [11] to observe the difference in the immune expression in colorectal cancer patients with and without tumor recurrence. As a result, 30 differentially expressed genes (DEGs) were identified (Figure S1). The expression differences of these RNAs in different samples are displayed in a heatmap in Figure S1. The 30 DEGs were incorporated as covariates into the CATCH model. Data were divided into the training set (67%) and the test set (33%), with the training set maintaining an original disease recurrence rate of 0.29. Performance measures were calculated using a testing set with 100 iterations. The performance of the proposed method was then compared with that of random forest (RF) and linear discriminant analysis (LDA), the most common algorithms in machine learning for classification. Table 1 shows the performance of the RF, LDA, and CATCH models (Table 1). The average AUCs for the CATCH, RF, and LDA models were 0.56, 0.46, and 0.46, respectively. The CATCH model had a high sensitivity of 0.66 and an F1 of 0.69 in the testing set. These results indicate that the CATCH model can integrate transformed radiomics-based CT images into RNA immune genomic expression data to achieve an accurate classification of recurrent CRC.

### 3.3. Adjusted Radiomic Features Obtained from CATCH Model for Cancer Recurrence

**Figure 2:**
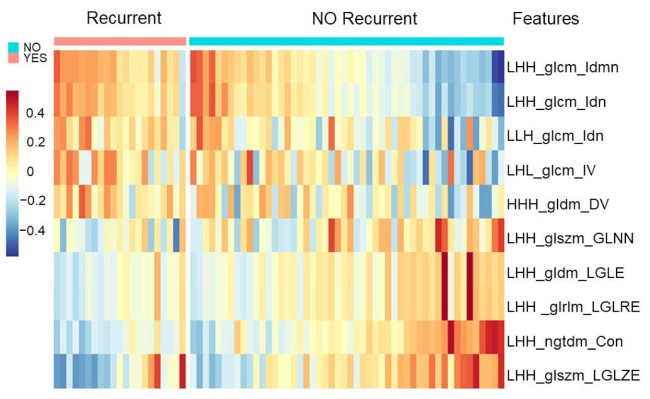
Adjusted radiomic features in the CATCH model. The 10 most significant adjusted radiomic features distinguish the recurrent CRC from the non-recurrent CRC. Positive coefficient values indicate higher significant association between radiomic features and recurrence. A more significant absolute value of the coefficient indicates more profound influence on recurrence.

The ten most significant adjusted radiomic features were selected based on the variable selection algorithm in the CATCH model to investigate the usefulness for predicting cancer recurrence (Table S5 and Figure 2). The adjusted radiomic features could clearly distinguish recurrent CRC from nonrecurrent CRC. The range of the coefficient $B_{k}$ was from 6.57 to −5.71 (Table 2). If the coefficient value was positive, greater significance of the variable was associated with the probability of CRC recurrence. If the absolute value of the coefficient was higher, its influence on recurrence was more remarkable. There were distinct radiomic profiles of the recurrent cancer patterns. Recurrence was positively correlated with wavelet.LHH_glcm_Idmn and wavelet.LHH_glcm_Idn. LHH_glcm_Idmn, which measures Idm (inverse difference moment or Homogeneity 2), measures the local homogeneity of an image. Idmn, local homogeneity, is associated with cancer recurrence. LHH_glcm_Idn, which measures the inverse normalized difference (Idn), is another measure of the local homogeneity of an image. Idn normalizes the difference between neighboring intensity values by dividing by the total number of discrete intensity values. Idn, local homogeneity, is associated with cancer recurrence. 

Meanwhile, recurrence was negatively correlated with wavelet LHH_glszm_LowGrayLevelZoneEmphasis (LGLZE), wavelet LHH_ngtdm_Contrast. LHH_glszm_LGLZE measures the distribution of lower gray-level size zones, with a higher value indicating a greater proportion of lower gray-level values and size zones in the image. LGLZE, such as tumor necrosis or mucinous lesions in CT scan images, is associated with cancer non-recurrence. LHH_ngtdm_Contrast measures the spatial intensity change but depends on the dynamic range of gray. In this study, the contrast was high when both the dynamic range and spatial change rate were high. Contrast imaging is associated with cancer non-recurrence (Table S6). These findings may provide significant adjusted radiomic features for predicting recurrence in stage III CRC.

### 3.4. Adjusted Radiomic Features Impact Clinical Outcome

**Figure 3:**
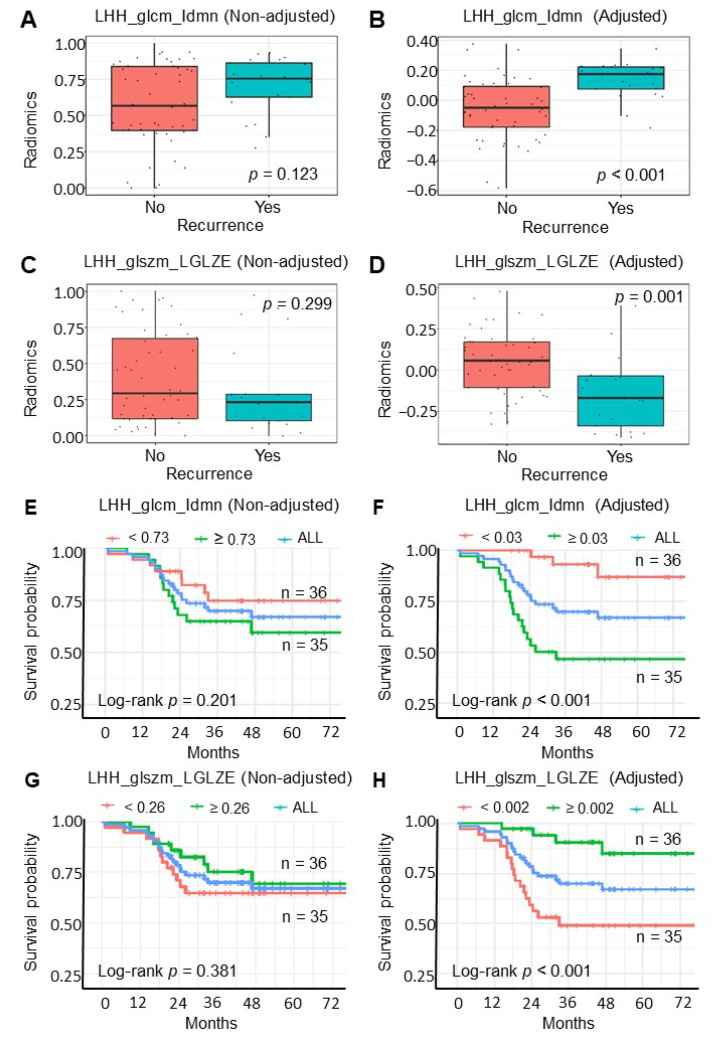
Boxplot and Kaplan–Meier survival curves comparing the risk of cancer recurrence and recurrence-free survival (RFS) among patients with adjusted radiomic features or without radiomic features. (**A**) Boxplot of cancer recurrence in patients without adjusted LHH_glcm_Idmn radiomic features. (**B**) Boxplot of cancer recurrence in patients with adjusted LHH_glcm_Idmn radiomic features. (**C**) Boxplot of cancer recurrence in patients without adjusted LHH_glszm_LGLZE radiomic features. (**D**) Boxplot of cancer recurrence in patients with adjusted LHH_glszm_LGLZE radiomic features. (**E**) Kaplan–Meier survival curves of recurrence-free survival by wavelet LHH_glcm_Idmn without adjusted radiomic features. (**F**) Kaplan–Meier survival curves of recurrence-free survival by wavelet LHH_glcm_Idmn with adjusted radiomic features. (**G**) Kaplan–Meier survival curves of recurrence-free survival by LHH_glszm_LGLZE without adjusted radiomic features. (**H**) Kaplan–Meier survival curves of recurrence-free survival by LHH_glszm_LGLZE with adjusted radiomic features. The blue curve represents overall population. The green curve represents the patients with radiomic data above the median. The red curve represents the patients with radiomic data below the median.

After comparison of the clinical outcomes of adjusted and non-adjusted radiomic features, only two non-adjusted radiomic features, namely LHL_glcm_InverseVariance (IV) and HHH_gldm_DependenceVariance (DV), were correlated with cancer recurrence (Figure S2). After adjusting radiomic features using the CATCH model, we obtained 10 significantly adjusted radiomic features correlated with cancer recurrence. Boxplot and Kaplan–Meier survival curves comparing the risk of cancer recurrence and recurrence-free survival (RFS) among patients with adjusted radiomic features or without radiomic features are shown in Figure S2. Figure 3A (non-adjusted, *p*-value = 0.123) and 3B (adjusted, *p*-value < 0.001) show the boxplot of wavelet LHH_glcm_Idmn. Figure 3C (non-adjusted, *p*-value = 0.299) and Figure 3D (adjusted, *p*-value = 0.001) show the boxplot of wavelet.LHH_glszm_LGLZE.

In addition to recurrence prediction, the survival impact of the adjusted radiomic features was compared with that of non-adjusted radiomic features. After adjusting for radiomic features using the CATCH model, seven significantly adjusted radiomic features (Figure S2) that correlated with cancer RFS were obtained. The non-adjusted LHH_glcm_Idmn (*p*-value = 0.201) (Figure 3E) was not a significant prognostic factor for RFS. In contrast, the adjusted LHH_glcm_Idmn was a significant prognostic factor for RFS (Figure 3F, *p*-value < 0.001). The non-adjusted LHH_glszm_LGLZE (*p*-value = 0.381) (Figure 3G) was not a significant prognostic factor for RFS, while the adjusted LHH_glcm_Idmn was a significant prognostic factor (Figure 3H, *p*-value < 0.001). These two adjusted radiomic features were associated with cancer recurrence and RFS. These results demonstrate the advantages of the CATCH model.

### 3.5. Correlation between Adjusted Radiomic Features and Immune Gene Expression

**Figure 4:**
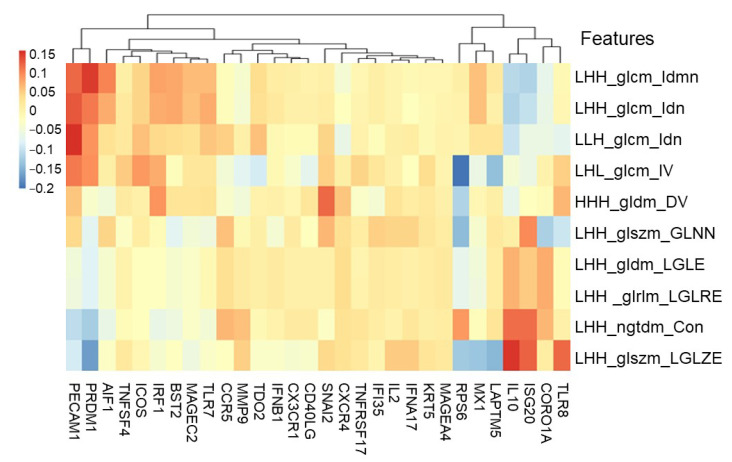
Heatmap visualization based on 10 significant adjusted radiomic features and immune gene expression. The correlation between adjusted radiomic features and immune gene expressions. The adjusted radiomic features are positively associated with the expression of the *PECAM1*, *PRDM1*, *AIF1*, *IL10*, *ISG20*, and *TLR8* genes.

The correlation between adjusted radiomic features of CT image data and tumor microenvironment-based immune gene expression in cancer tissues was investigated to identify potential therapeutic targets in high-risk CRC stage III patients. As shown in Figure 4 and Table S7, the spectrum of RNA expression was identified in the 10 significantly adjusted radiomic features. The adjusted radiomic features were positively associated with the expression of *PECAM1*, *PRDM1*, *AIF1*, IL10, *ISG20*, and *TLR8*. For recurrence, the highest positive correlation was found between the adjusted radiomic features LHH_glcm_Idmn, LHH_glcm_Idn, and LLH_glcm_Idn and expressions of immune genes *PECAM1*, *PRDM1*, and *AIF1*. For non-recurrence, the highest positive correlation was found between the adjusted radiomic feature of LHH_glszm_LGLZE and LHH_ngtdm_Contrast and the expression of the immune genes *IL10*, *ISG20*, and *TLR8*. *PECAM1* and *PRDM1* immune gene expression was negatively related to the non-cancer recurrence-adjusted radiomic features of LHH_glszm_LGLZE and LHH_ngtdm_Contrast. These results indicate that potential therapeutic targets, such as *PECAM1*, *PRDM1*, and *AIF1*, can be identified by adjusting the radiomic features that impact cancer. 

### 3.6. PECAM1 as a Therapeutic Target Identified by Adjusted Radiomic Features in Recurrent Colorectal Cancer

**Figure 5:**
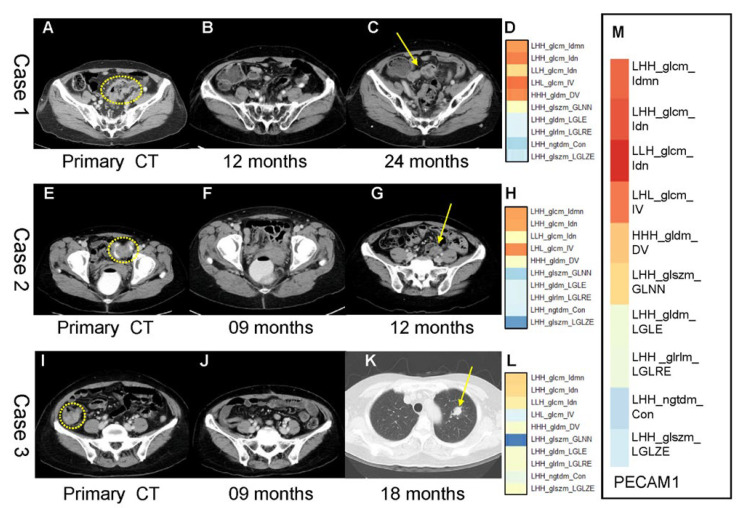
Treatment strategies by adjusted radiomic features in recurrent colorectal cancer. Three stage III CRC patients with individualized treatment targets identified by adjusted radiomic features. *PECAM1* is indicated as the potential therapeutic target.

Figure 5 shows three patients with individualized therapeutic targets based on adjusted radiomic features. The integration of specific immune gene expression and radiomic features provides valuable information for clinical decision-making and guidance for individualized therapeutic target options. Based on the association of immune gene expression and adjusted CT image-derived radiomic features, which further correlate with recurrence in stage III CRC patients, some examples of the applications of our model are provided.

The first patient (Figure 5A) was a 61-year-old woman with pathological stage III left-sided colon cancer at the initial diagnosis. The patient underwent standard surgical resection followed by adjuvant chemotherapy with modified FOLFOX (mFOLFOX7) for 12 cycles (Figure 5A,B). Multiple peritoneal metastases were detected on CT at 24 months postoperatively (Figure 5C). The feature profile showed higher LHH_glcm_Idmn, LHH_glcm_Idn, LHL_glcm_IV, and HHH_gldm_DV values, indicating a higher risk of recurrence (Figure 5D). These adjusted features correlated with *PECAM1* and *SNAI2* RNA expression (Figure 5M). Therefore, it may be a therapeutic target.

The second patient (Figure 5E) was a 60-year-old woman with pathological stage III sigmoid colon cancer at the time of the initial diagnosis. The patient underwent standard surgical resection followed by adjuvant chemotherapy with mFOLFOX7 for 12 cycles (Figure 5F). Recurrence of lymph nodes was detected by CT at 12 months postoperatively (Figure 5G). The feature profile showed higher LHH_glcm_Idmn, LHH_glcm_Idn, and LHL_glcm_IV values, indicating a higher risk of recurrence (Figure 5H). These adjusted features correlated with *PECAM1* RNA expression.

The third patient (Figure 5I) was a 76-year-old woman with pathological stage III right-sided colon cancer at the time of initial diagnosis. The patient underwent standard surgical resection followed by adjuvant chemotherapy with mFOLFOX7 for 12 cycles (Figure 5I,J). A single lung metastasis was detected on CT at 12 months postoperatively (Figure 5K). They were not correlated with the adjusted radiomic features profile, indicating a borderline risk of recurrence (Figure 5L).

## 4. Discussion

CRC is an etiologically heterogeneous disease that involves several distinct biological pathways and CT scan presentations. This study used diagnostic CT images and gene expression as covariates in the CATCH model to predict recurrence in CRC patients. The results show that the model predicts recurrence by adjusting radiomic features and identifies potential therapeutic targets in CRC. Our results highlight the following important points. First, the CATCH model efficiently integrates high-dimensional radiomic features and covariates of immune gene expression to predict cancer recurrence in small datasets. Second, 10 textural associated adjusted radiomic features are selected for cancer recurrence, with 7 of these adjusted radiomic features being associated with RFS. Finally, we established a correlation between radiomic features and immune gene expression, providing biological relevance and individualized therapeutic targets for patients with recurrence.

CT-based radiomic signatures are potential biomarkers for predicting CRC recurrence [1]. Prognosis prediction based on informative DEGs also has higher predictive accuracy for CRC prognosis. However, few studies have attempted to analyze immune gene expression as a covariate for high-dimensional image information. Further, the sample is often limited to surveys collecting both CT scan image data and gene expression levels due to the high intrinsic cost of data collection involving human participants. In such conditions, the machine learning algorithms could have poor accuracy because the learning algorithm does not have enough data to learn from.

The CATCH model solves high-dimensional data and feature integration strategies from disparate sources and unbalanced datasets. Furthermore, it incorporates a feature-selection algorithm into the classification model. Thus, the CATCH model can achieve acceptable accuracy with limited data and covariate adjustments. We used this advantage in the current study. To establish a model to manage high-dimensional image data, the 1037 radiomic feature data were converted into 33×32-pixel image data, representing a transformed image, using a feature extraction algorithm. Then, we integrated 30 DEGs and 33×32-pixel image data to accurately predict cancer recurrence. In the traditional LDA and RF methods, a high number of features, 1037 high-dimensional features plus 30 DEGs, and unbalanced datasets were a problem for selecting the essential features. These machine-learning models struggled to integrate data from disparate sources and unbalanced datasets. LDA and RF models also had poorer sensitivity. Because the sample size is small, these machine-learning models were utilized without tuning processes to prevent overfitting. Although the performance of these machine-learning methods could be improved, it cannot offer interpretable discriminant analysis results. The study goal was to utilize the CATCH model to predict the risk of cancer recurrence and provide therapeutic strategies in our colorectal cancer patients. Using radiomic biomarkers and RNA expression data, we have provided a useful clinical model that will help physicians to make better-informed decisions regarding the short-interval CT scan follow-ups and potential drug targets. Collectively, these results indicate that the CATCH model can efficiently integrate high-dimensional radiomic features and covariates to predict cancer recurrence in small datasets.

Immune gene expression involves both clinical outcomes and radiomic expression. Previous studies have explored radiomic signatures and specific gene expression. CT scan-based radiomic features have been found to be significantly associated with *KRAS* or *BRAF* mutations [12,13]. A study in France demonstrated an association between gene expression and radiomics; for example, *ABCC2* expression was correlated with LGLZE and SZLGE [14]. In addition, radiomic features and gene expression of *ABCC2* have been identified as prognostic factors for survival [14]. Our model adopted gene expression as an additional covariate to improve the predictive accuracy for clinical outcomes. The performance of the CATCH model showed that radiomic features adjusted by immune gene expression are good prognostic factors for cancer recurrence. No prognostic risk factors were identified in clinicopathological and genetic features (Table S3). Therefore, we did not apply the clinicopathological and genetic features for risk modeling. Of all 71 CRC patients, there was no significant difference in genetic profiling including MMR, KRAS, and BRAF status between patients with and without cancer recurrence. The median age of these patients was 58 years. We did not identify clinically important factors such as age or pathological stage affecting cancer recurrence by statistical analysis (Tables S3 and S4).

Tumor heterogeneity, which is closely reflected in imaging data, is an important indicator of tumor growth and metastasis. In this study, 2/10 non-adjusted radiomic features were associated with cancer recurrence. After adjusting the covariant from the TME-associated immune gene expression, 10 adjusted radiomic features associated were identified to be associated with recurrence. There were no radiomic features associated with first-order statistics, with most features related to textural features. In the radiomic prognostic vector of ovarian cancer, the authors discovered and validated prognostic imaging value. They associated these findings with stromal biological factors [15]. Similarly, our results also indicate that stromal heterogeneity of images plays a prognostic role in cancer recurrence. Seven adjusted radiomic features were related not only to recurrence risk, but also survival. Textural wavelet decomposition was found to affect the prognosis of stage III CRC.

By integrating clinical and radiomic features, a clinical radiomics-based model accurately predicted recurrence in patients with stage II CRC [5,14], supporting the idea that clinical and radiomic signatures can serve as markers for survival stratification. Our study balanced the clinical features of patients with recurrent and nonrecurrent stage III cancer. These clinicopathological risk factors, including tumor invasion stage and lymph node stage, lack accuracy to identify patients at high risk of recurrence. Multivariate survival analysis showed that the clinical factors were not significantly associated with survival. Several previous studies have attempted to develop prognostic tools based on the radiomics-only model. However, these prognostic models are challenging to apply in routine clinical practice because of the lack of association between image features and molecular biology. Our model provides further guidance for individualized treatment according to the associated immune gene expression for patients at high risk of recurrence. *PECAM1*, platelet endothelial cell adhesion molecule 1 (*CD31*), is involved in tumor angiogenesis and endothelial cell migration [16]. The protein encoded by the PECAM1 gene is an endothelial cell marker used to evaluate tumor microvessels and vascular density. Previous studies have demonstrated that a high tumor microvessel count and density predict CRC recurrence and overall survival [17,18,19]. CRC patients with high *CD31* expression have poor prognosis [19]. Given that *PECAM1* contributes to colorectal peritoneal metastasis, it may be a potential therapeutic target for CRC [20,21].

There are some limitations in our study. In a clinical setting, we often only have a small dataset to work with. For example, RNA sequencing is still expensive and time-consuming for the sequencing process. It is not easy for cancer patients to have both CT scan and RNA sequencing datasets. Secondly, the result of AUC is rather low in our study. Our prediction model was designed to assist healthcare professionals and patients with decisions about the short interval CT scan surveillance in clinical practice. The clinical risk of misclassification is increased with the radiation exposure of CT scans in cancer patients. Following the National Comprehensive Cancer Network (NCCN) guidelines [22], the stage III CRC patients received standard 6–12 months CT scan follow-ups. We could improve the cancer outcome by the early diagnosis of resectable CRC with lung or liver metastasis based on short-interval CT scan follow-ups (e.g., < 6 months CT scan interval for early detection of cancer metastasis). Third, many algorithms, such as the Synthetic Minority Oversampling Technique (SMOTE) [23], could also improve the prediction accuracy of imbalanced data and small datasets. The combat harmonization method has been adapted to neuroimaging studies with data heterogeneity [24]. However, only the CATCH model could produce interpretable discriminate results that can be used to identify potential therapeutic targets. It is worth noting that the CATCH model is developed under the assumption of homogeneity in data. In the future, it will be interesting to develop a modified CATCH model for heterogeneity data.

## 5. Conclusions

Our CATCH model efficiently adjusted high-dimensional radiomic features with covariates of immune gene expression to predict cancer recurrence. The correlation between radiomic features and immune gene expression may be biologically relevant. The adjusted radiomic features associated with recurrence in our model provide a basis for individualized treatment in stage III CRC.

## Figures and Tables

**Table 1:** Performance of machine learning models.

Methods	Accuracy	Sensitivity	Specificity	F1 score	AUC
Random forest	0.68	0.16	0.83	0.24	0.46
LDA	0.64	0.32	0.78	0.35	0.55
CATCH	0.60	0.66	0.48	0.69	0.56

Abbreviations: LDA, linear discriminant analysis; CATCH, covariate-adjusted, proposed tensor classification in high dimensions; AUC, area under the curve.

**Table 2:** Coefficients of adjusted radiomic features.

Features	Coefficient
wavelet LHH_glcm_Idmn	6.57
wavelet LHH_glcm_Idn	4.45
wavelet LLH_glcm_Idn	0.69
wavelet LHL_glcm_InverseVariance (IV)	0.07
wavelet HHH_gldm_DependenceVariance (DV)	0.06
wavelet LHH_glszm_GrayLevelNonUniformityNormalized (GLNN)	−0.11
wavelet LHH_gldm_LowGrayLevelEmphasis (LGLE)	−0.20
wavelet LHH_glrlm_LowGrayLevelRunEmphasis (LGLRE)	−0.73
wavelet LHH_ngtdm_Contrast	−5.22
wavelet LHH_glszm_LowGrayLevelZoneEmphasis (LGLZE)	−5.71

## Data Availability

The datasets used and analyzed during the current study are available from the corresponding author on reasonable request, and supplementary information files are available for this manuscript.

## Supplementary Materials

The following are available online at https://www.mdpi.com/article/10.3390/cancers14081895/s1. Figure S1: Heatmap of significant differentially expressed genes (DEGs) and clinical outcome; Figure S2: Boxplot and Kaplan–Meier survival curves comparing the risk of cancer recurrence and recurrence-free survival (RFS) among patients with adjusted radiomic features or without radiomic features; Table S1: Wavelets and LoG features per patient; Table S2: The spectrum of 1037 radiomic features; Table S3: Patients’ characteristics in recurrent and non-recurrent groups; Table S4: The clinical and genetic features in cancer patients; Table S5: The adjusted radiomic features in cancer patients; Table S6: The clinical impact of adjusted radiomic features; Table S7: The correlation of adjusted radiomics and RNA expression.

## Abbreviations

AUC	area under the curve
CATCH	covariate-adjusted tensor classification in the high-dimension
CRC	colorectal cancer
CT	computed tomography
CRC	colorectal cancer
DEGs	differentially expressed genes
DICOM	Digital Imaging and Communications in Medicine
DV	dependence variance
FOLFOX	leucovorin (folinic acid), fluorouracil, and oxaliplatin
IV	inverse variance
LDA	linear discriminant analysis
LGLZE	low gray level zone emphasis
mFOLFOX7	modified FOLFOX
MMR	mismatch repair
NCCN	National Comprehensive Cancer Network
NCKUH	National Cheng Kung University Hospital
PACS	picture archiving and communication system
RF	random forest
RFS	recurrence-free survival
TME	tumor microenvironment
VOI	volume of interest
