# Supplementary material for: Adjusted CT Image-Based Radiomic Features Combined with Immune Genomic Expression Achieve Accurate Prognostic Classification and Identification of Therapeutic Targets in Stage III Colorectal Cancer
Source: Cancers (Basel). 2022 Apr 8;14(8):1895. doi: 10.3390/cancers14081895 (PMC9029745; doi:10.3390/cancers14081895)
Supplement: Supplementary file 1 [file cancers-14-01895-s001.zip › Supplementary Figure S1. Hemap of DEG20220214.pdf]

Figure S1

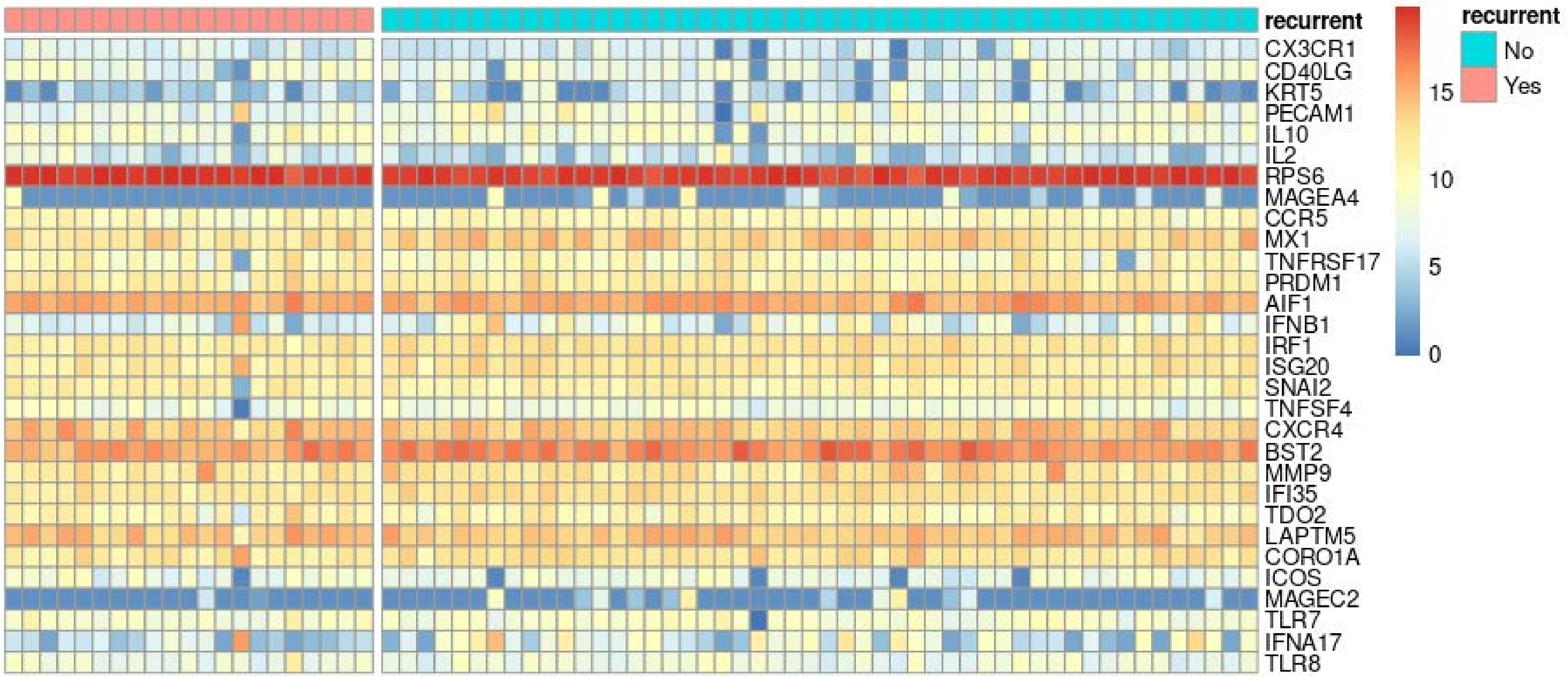

Supplementary Figure S1. Heatmap of significant differentially expressed genes (DEGs) and clinical outcome. 30 significant differentially expressed genes (DEGs) were selected from 398 RNA genes by cancer recurrence (n=21) or non-cancer recurrence (n=50).
