# Supplementary material for: Adjusted CT Image-Based Radiomic Features Combined with Immune Genomic Expression Achieve Accurate Prognostic Classification and Identification of Therapeutic Targets in Stage III Colorectal Cancer
Source: Cancers (Basel). 2022 Apr 8;14(8):1895. doi: 10.3390/cancers14081895 (PMC9029745; doi:10.3390/cancers14081895)
Supplement: Supplementary file 1 [file cancers-14-01895-s001.zip › Supplementary Figure S2. Boxplot and Kaplan-Meier survival curves.pdf]

Supplementary Figure S2. Boxplot : Non-adjusted vs adjusted (Left : Non-adjusted ; Right : adjusted)

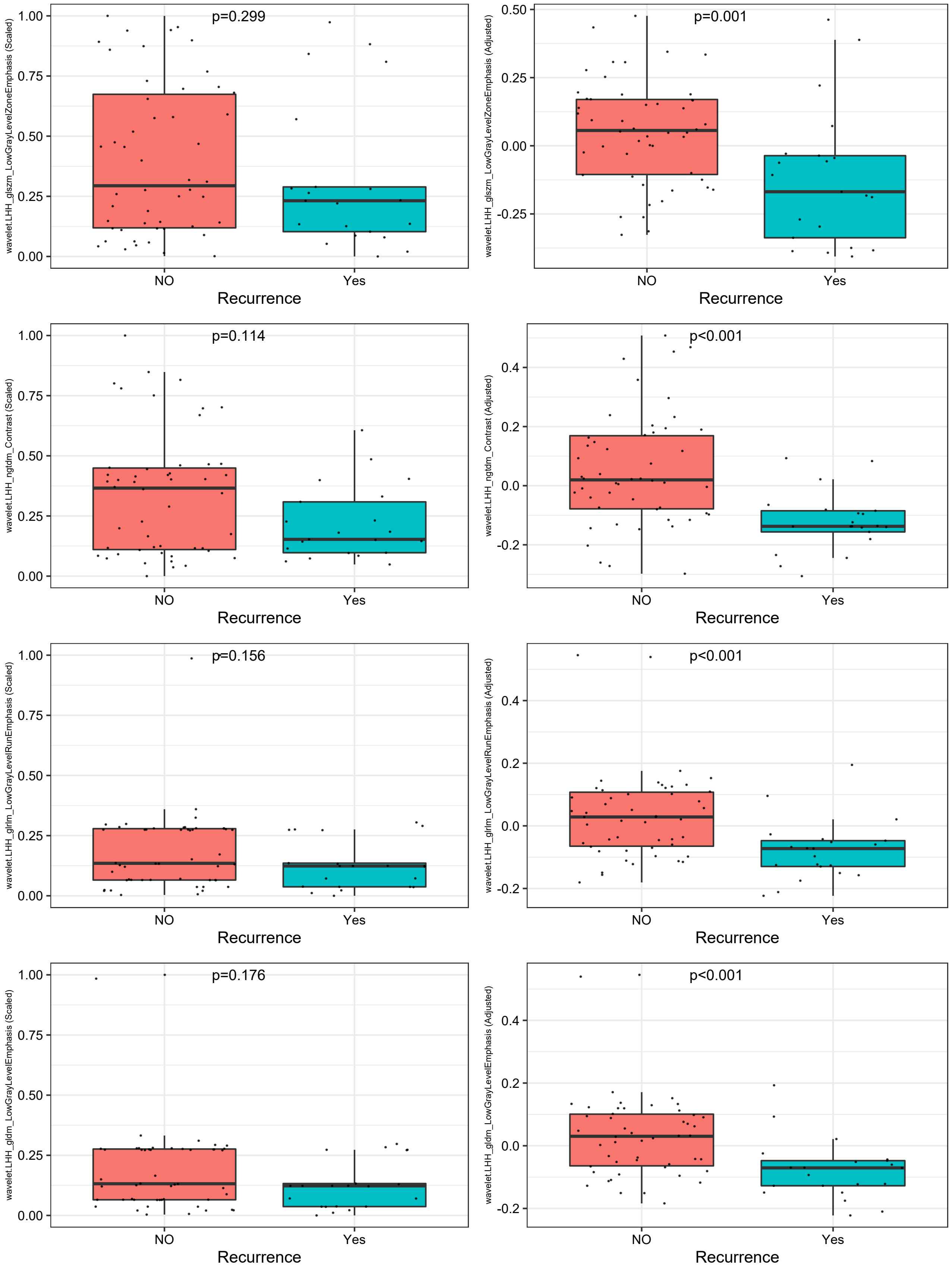

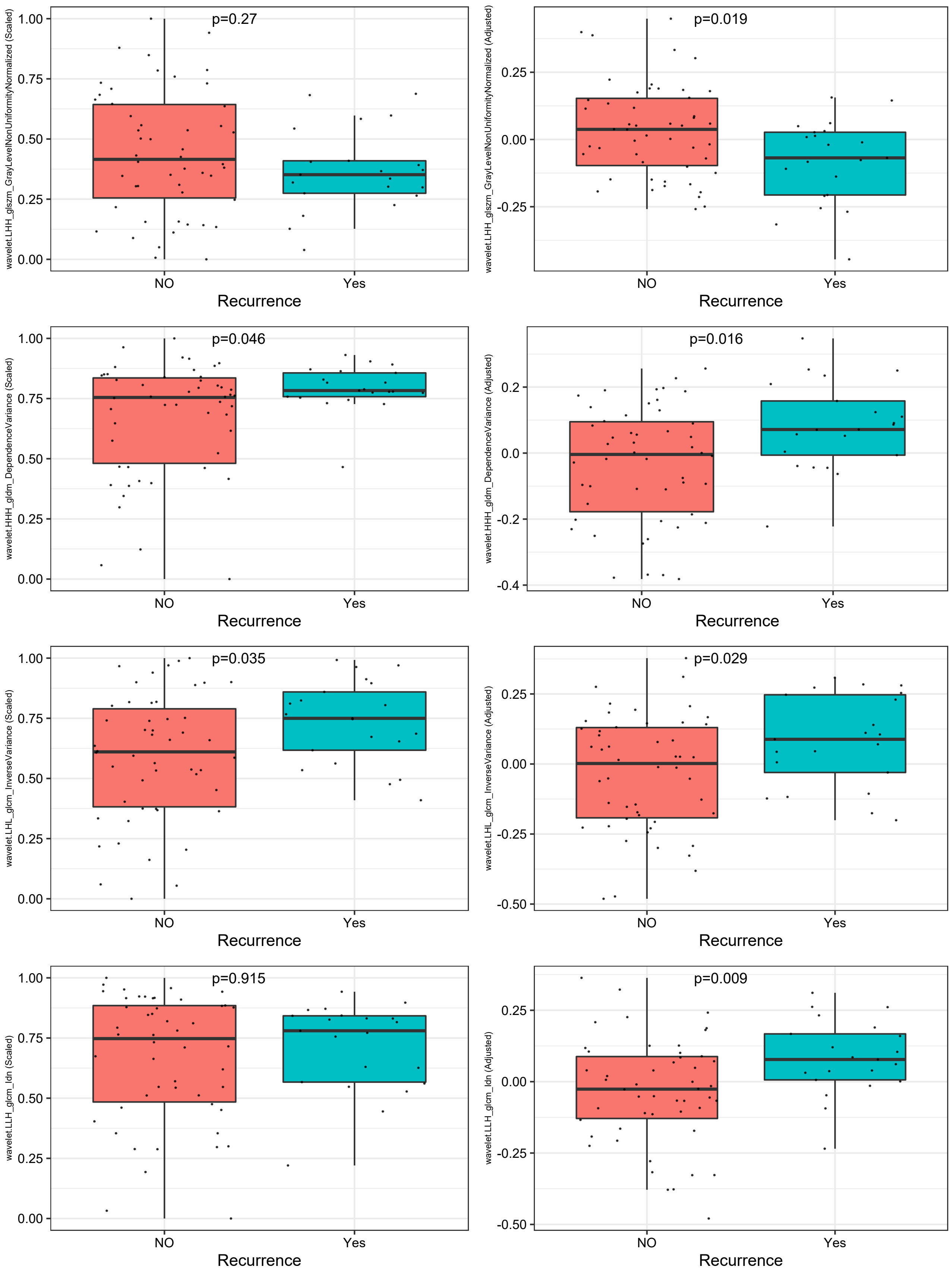
wavelet.LHH_glszm_GrayLevelNonUniformityNormalized (Scaled)
p=0.27
1.00
0.75
0.50
0.25
0.00
NO
Yes
Recurrence
wavelet.LHH_glszm_GrayLevelNonUniformityNormalized (Adjusted)
p=0.019
0.25
0.00
-0.25
NO
Yes
Recurrence
wavelet.HHH_gldm_DependenceVariance (Scaled)
p=0.046
1.00
0.75
0.50
0.25
0.00
NO
Yes
Recurrence
wavelet.HHH_gldm_DependenceVariance (Adjusted)
p=0.016
0.2
0.0
-0.2
-0.4
NO
Yes
Recurrence
wavelet.LHL_glcm_InverseVariance (Scaled)
p=0.035
1.00
0.75
0.50
0.25
0.00
NO
Yes
Recurrence
wavelet.LHL_glcm_InverseVariance (Adjusted)
p=0.029
0.25
0.00
-0.25
-0.50
NO
Yes
Recurrence
wavelet.LLH_glcm_Idn (Scaled)
p=0.915
1.00
0.75
0.50
0.25
0.00
NO
Yes
Recurrence
wavelet.LLH_glcm_Idn (Adjusted)
p=0.009
0.25
0.00
-0.25
-0.50
NO
Yes
Recurrence

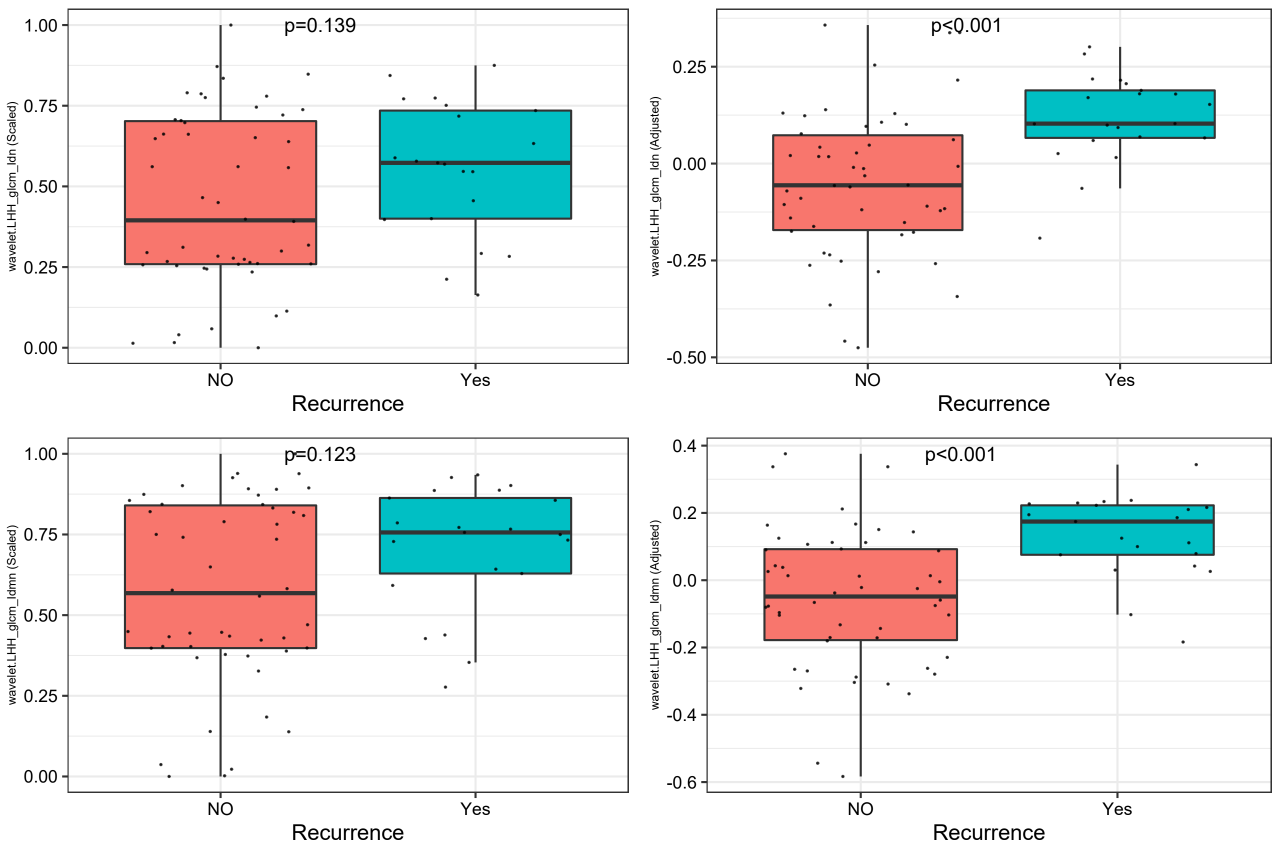
p=0.139
wavelet.LHH_glcm_Idn (Scaled)
NO
Yes
Recurrence
p<0.001
wavelet.LHH_glcm_Idn (Adjusted)
NO
Yes
Recurrence
p=0.123
wavelet.LHH_glcm_Idmn (Scaled)
NO
Yes
Recurrence
p<0.001
wavelet.LHH_glcm_Idmn (Adjusted)
NO
Yes
Recurrence

Kaplan-Meier curve: Non-adjusted vs adjusted (Left : Non-adjusted ; Right : adjusted)

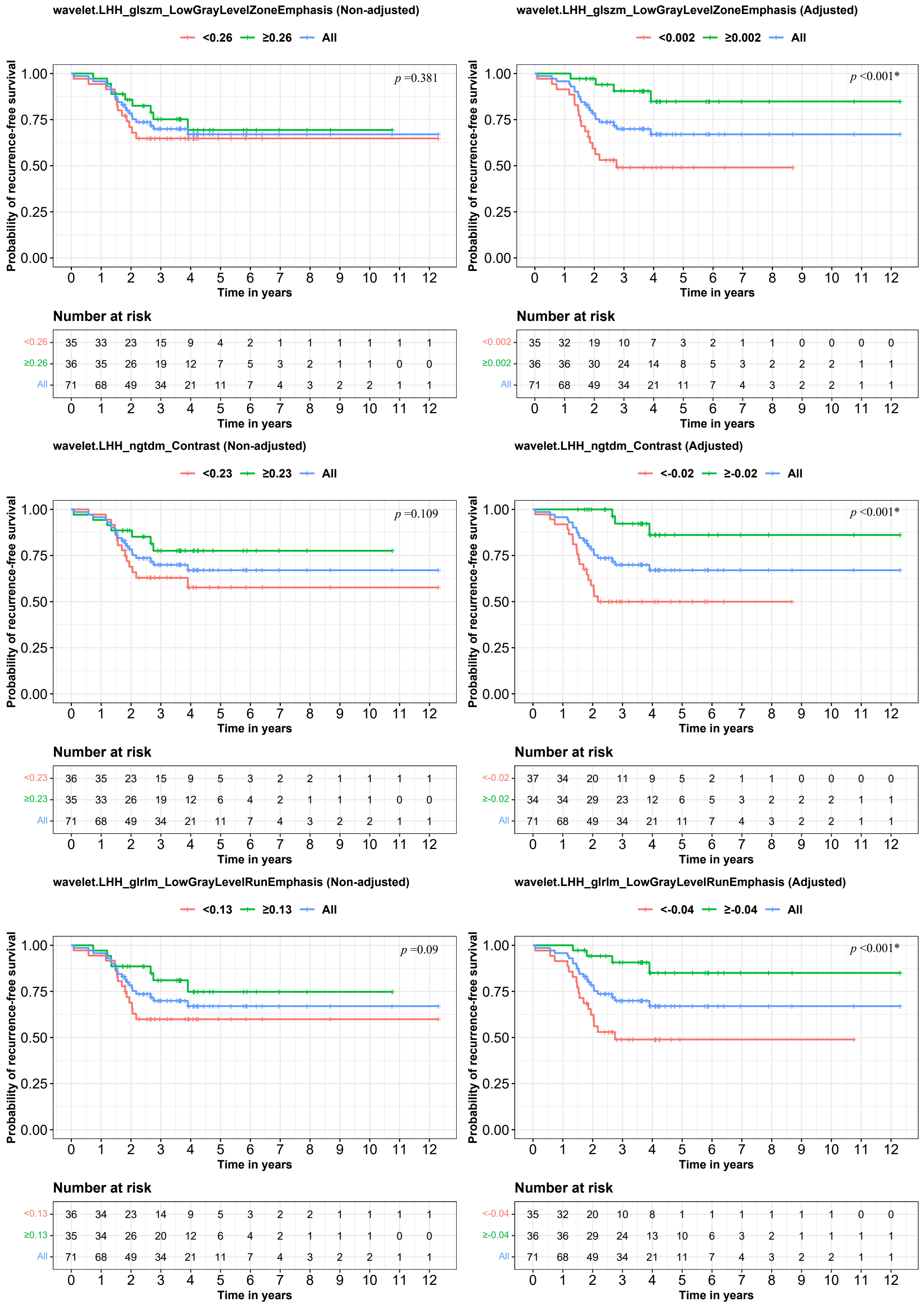

wavelet.LHH_gldm_LowGrayLevelEmphasis (Non-adjusted)

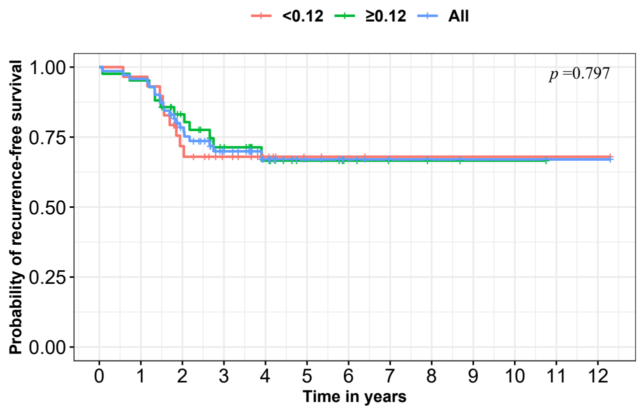

Number at risk

	0	1	2	3	4	5	6	7	8	9	10	11	12
<0.12	29	28	19	13	7	3	2	1	1	1	1	1	1
≥0.12	42	40	30	21	14	8	5	3	2	1	1	0	0
All	71	68	49	34	21	11	7	4	3	2	2	1	1

Time in years

wavelet.LHH_gldm_LowGrayLevelEmphasis (Adjusted)

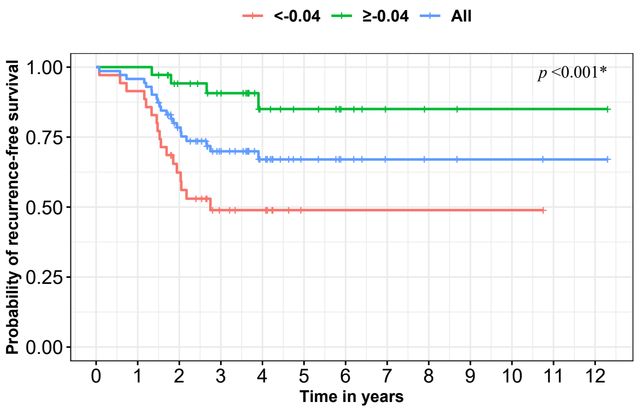

Number at risk

	0	1	2	3	4	5	6	7	8	9	10	11	12
<-0.04	35	32	20	10	8	1	1	1	1	1	1	0	0
≥-0.04	36	36	29	24	13	10	6	3	2	1	1	1	1
All	71	68	49	34	21	11	7	4	3	2	2	1	1

Time in years

wavelet.LHH_glszm_GrayLevelNonUniformityNormalized (Non-adjusted)

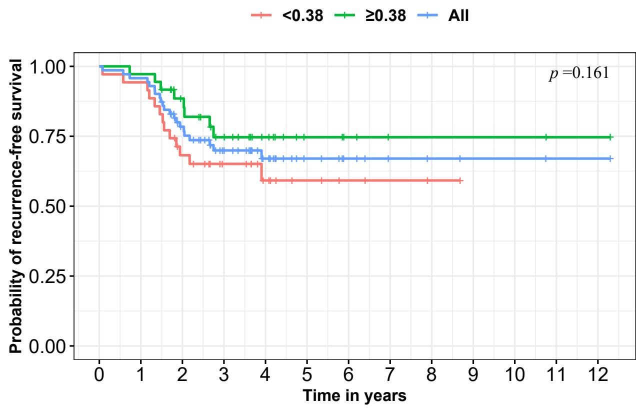

Number at risk

	0	1	2	3	4	5	6	7	8	9	10	11	12
<0.38	35	33	22	15	9	5	3	2	1	0	0	0	0
≥0.38	36	35	27	19	12	6	4	2	2	2	2	1	1
All	71	68	49	34	21	11	7	4	3	2	2	1	1

Time in years

wavelet.LHH_glszm_GrayLevelNonUniformityNormalized (Adjusted)

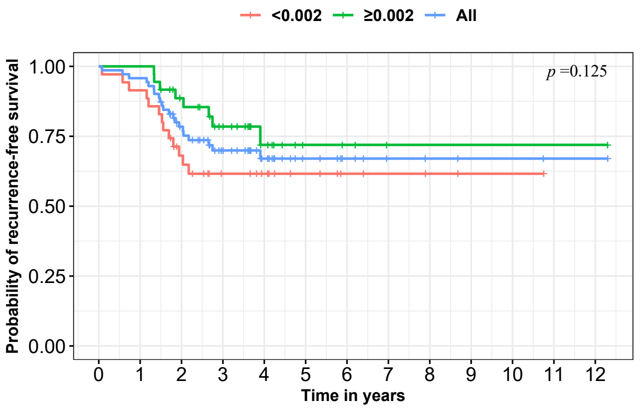

Number at risk

	0	1	2	3	4	5	6	7	8	9	10	11	12
<0.002	35	32	21	14	11	7	4	3	2	1	1	0	0
≥0.002	36	36	28	20	10	4	3	1	1	1	1	1	1
All	71	68	49	34	21	11	7	4	3	2	2	1	1

Time in years

wavelet.HHH_gldm_DependenceVariance (Non-adjusted)

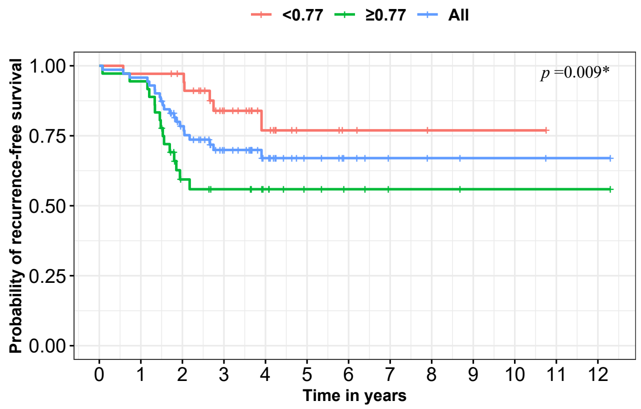

Number at risk

	0	1	2	3	4	5	6	7	8	9	10	11	12
<0.77	35	34	32	20	11	5	3	2	1	1	1	0	0
≥0.77	36	34	17	14	10	6	4	2	2	1	1	1	1
All	71	68	49	34	21	11	7	4	3	2	2	1	1

Time in years

wavelet.HHH_gldm_DependenceVariance (Adjusted)

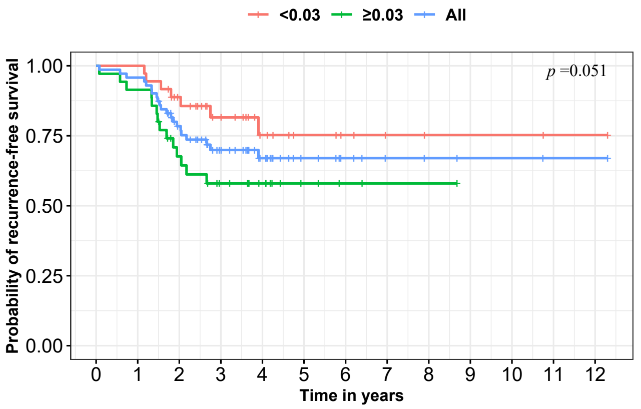

Number at risk

	0	1	2	3	4	5	6	7	8	9	10	11	12
<0.03	36	36	28	19	11	7	5	3	2	2	2	1	1
≥0.03	35	32	21	15	10	4	2	1	1	0	0	0	0
All	71	68	49	34	21	11	7	4	3	2	2	1	1

Time in years

wavelet.LHL_glcm_InverseVariance (Non-adjusted)

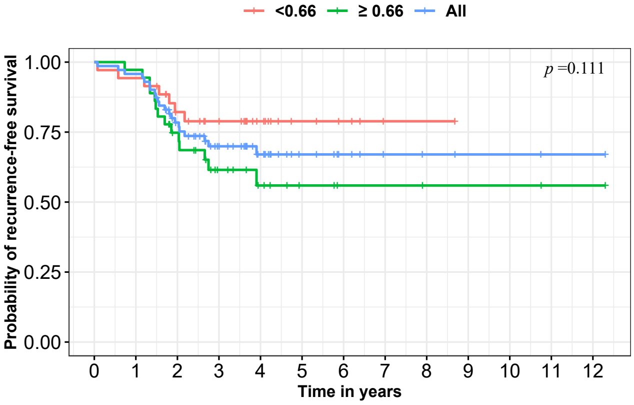

Number at risk

	0	1	2	3	4	5	6	7	8	9	10	11	12
<0.66	35	33	25	20	12	6	4	1	1	0	0	0	0
≥ 0.66	36	35	24	14	9	5	3	3	2	2	2	1	1
All	71	68	49	34	21	11	7	4	3	2	2	1	1

Time in years

wavelet.LHL_glcm_InverseVariance (Adjusted)

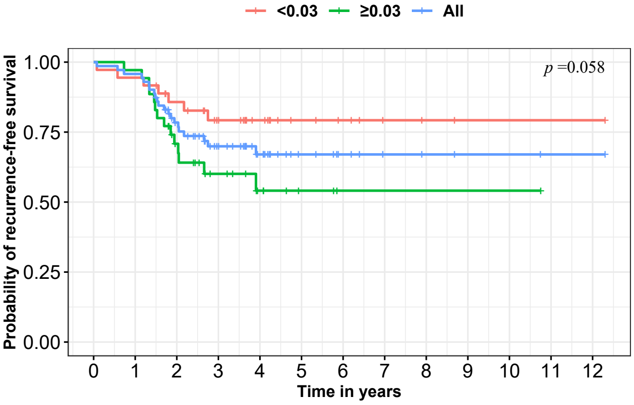

Number at risk

	0	1	2	3	4	5	6	7	8	9	10	11	12
<0.03	36	34	28	21	14	8	6	3	2	1	1	1	1
≥0.03	35	34	21	13	7	3	1	1	1	1	1	0	0
All	71	68	49	34	21	11	7	4	3	2	2	1	1

Time in years

wavelet.LLH_glcm_Idn (Non-adjusted)

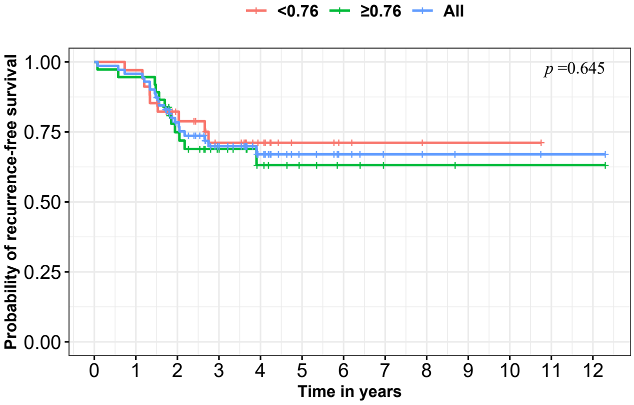

Number at risk

	0	1	2	3	4	5	6	7	8	9	10	11	12
<0.76	34	33	24	17	11	5	3	2	1	1	1	0	0
≥0.76	37	35	25	17	10	6	4	2	2	1	1	1	1
All	71	68	49	34	21	11	7	4	3	2	2	1	1

Time in years

wavelet.LLH_glcm_Idn (Adjusted)

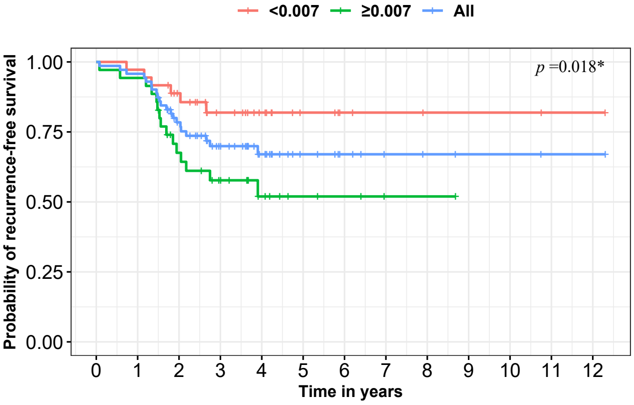

Number at risk

	0	1	2	3	4	5	6	7	8	9	10	11	12
<0.007	36	35	28	19	13	7	4	3	2	2	2	1	1
≥0.007	35	33	21	15	8	4	3	1	1	0	0	0	0
All	71	68	49	34	21	11	7	4	3	2	2	1	1

Time in years

wavelet.LHH_glcm_Idn (Non-adjusted)

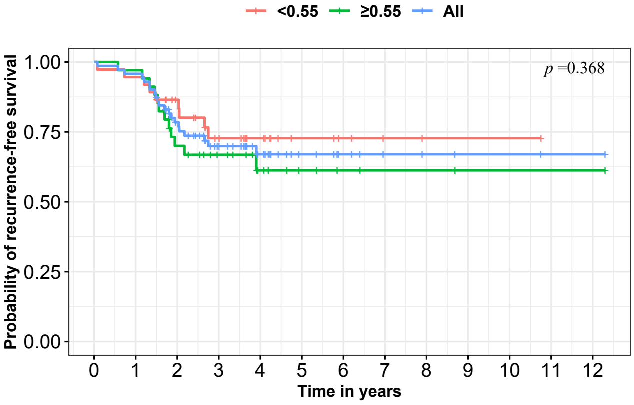

Number at risk

	0	1	2	3	4	5	6	7	8	9	10	11	12
<0.55	37	35	27	18	12	6	4	2	1	1	1	0	0
≥0.55	34	33	22	16	9	5	3	2	2	1	1	1	1
All	71	68	49	34	21	11	7	4	3	2	2	1	1

Time in years

wavelet.LHH_glcm_Idn (Adjusted)

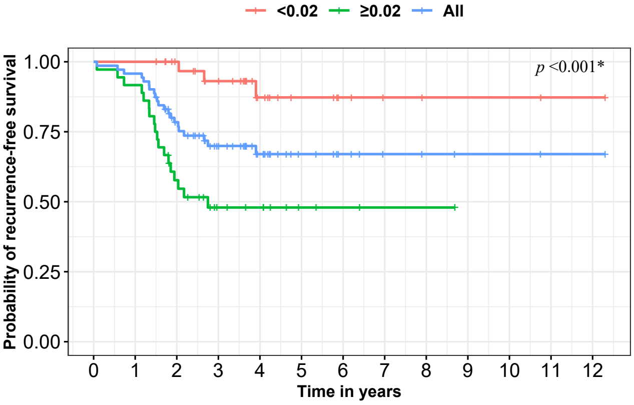

Number at risk

	0	1	2	3	4	5	6	7	8	9	10	11	12
<0.02	35	35	30	24	13	8	5	3	2	2	2	1	1
≥0.02	36	33	19	10	8	3	2	1	1	0	0	0	0
All	71	68	49	34	21	11	7	4	3	2	2	1	1

Time in years

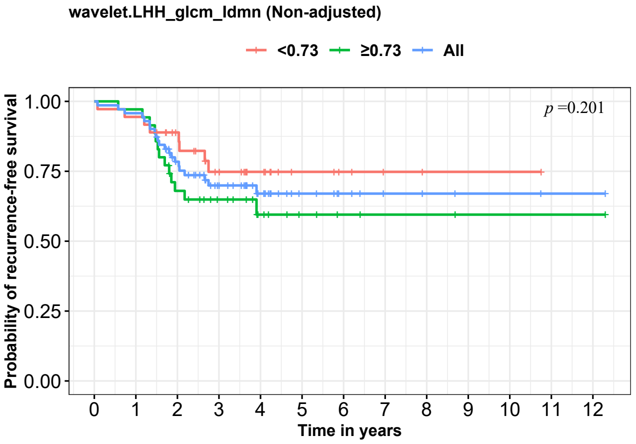

Number at risk

	0	1	2	3	4	5	6	7	8	9	10	11	12
<0.73	36	34	27	18	12	6	4	2	1	1	1	0	0
≥0.73	35	34	22	16	9	5	3	2	2	1	1	1	1
All	71	68	49	34	21	11	7	4	3	2	2	1	1

Time in years

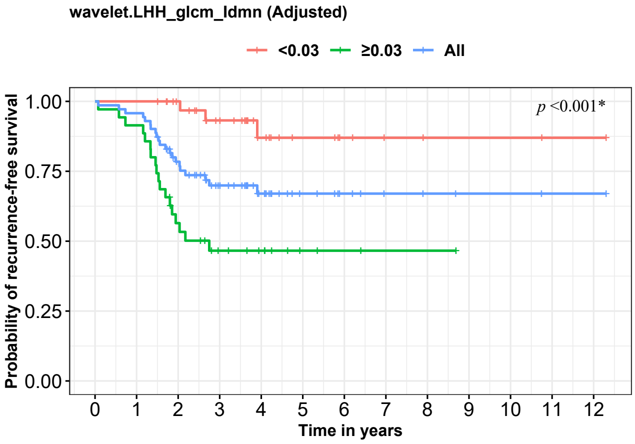

Number at risk

	0	1	2	3	4	5	6	7	8	9	10	11	12
<0.03	36	36	31	23	13	8	5	3	2	2	2	1	1
≥0.03	35	32	18	11	8	3	2	1	1	0	0	0	0
All	71	68	49	34	21	11	7	4	3	2	2	1	1

Time in years
