# Supplementary material for: Adjusted CT Image-Based Radiomic Features Combined with Immune Genomic Expression Achieve Accurate Prognostic Classification and Identification of Therapeutic Targets in Stage III Colorectal Cancer
Source: Cancers (Basel). 2022 Apr 8;14(8):1895. doi: 10.3390/cancers14081895 (PMC9029745; doi:10.3390/cancers14081895)
Supplement: Supplementary file 1 [file cancers-14-01895-s001.zip › Supplementary Table S1. Wavelets and LoG features per patient 0327.pdf]

Supplementary Table S1. Wavelets and LoG features per patient

Characteristic	Original	Wavelets transformation	Log-sigma-3-0-mm-3D	Log-sigma-5-0-mm-3D	All
First order	18	144	18	18	198
Shape-Related	14	-	-	-	14
GLCM	24	192	24	24	264
GLDM	14	112	14	14	154
GLRLM	16	128	16	16	176
GLSZM	16	128	16	16	176
NGTDM	5	40	5	5	55
Total	107	744	93	93	1037
