# Supplementary material for: Adjusted CT Image-Based Radiomic Features Combined with Immune Genomic Expression Achieve Accurate Prognostic Classification and Identification of Therapeutic Targets in Stage III Colorectal Cancer
Source: Cancers (Basel). 2022 Apr 8;14(8):1895. doi: 10.3390/cancers14081895 (PMC9029745; doi:10.3390/cancers14081895)
Supplement: Supplementary file 1 [file cancers-14-01895-s001.zip › Supplementary Table S3. Patientsía characteristics in recurrent and non-recurrent groups 20220326.pdf]

**Supplementary Table S3. Patients' characteristics in recurrent and non-recurrent groups**

| **Characteristic** | **Non-Recurrence (n = 50)** | **Recurrence (n = 21)** | ***p*-value** |
|---|---|---|---|
| Age | | | 0.395 |
| <65 | 37(74%) | 13(62%) | |
| >=65 | 13(26%) | 8(38%) | |
| Gender | | | 0.99 |
| Male | 25(50%) | 10(48%) | |
| Female | 25(50%) | 11(52%) | |
| Tumor site | | | 0.99 |
| Left | 39(78%) | 17(81%) | |
| Right | 11(22%) | 4(19%) | |
| Stage | | | 0.99 |
| II | 3(6%) | 1(5%) | |
| III | 47(94%) | 20(95%) | |
| Tumor invasion[a] | | | 0.716 |
| T1/T2 | 7(14%) | 2(10%) | |
| T3/T4 | 43(86%) | 19(90%) | |
| Lymph node[a] | | | 0.395 |
| N0/N1 | 37(74%) | 13(62%) | |
| N2 | 13(26%) | 8(38%) | |
| Mismatch Repair | | | |
| Loss | 5 | 0 | 0.065 |
| Preserved | 39 | 21 | |
| BRAF | | | 0.99 |
| Mutation | 3 | 1 | |
| Wild Type | 47 | 20 | |
| KRAS | | | 0.35 |
| Mutation | 17 | 11 | |
| Wild Type | 30 | 9 | |

Abbreviations: a. The American Joint Committee on Cancer Stage
