# Supplementary material for: Adjusted CT Image-Based Radiomic Features Combined with Immune Genomic Expression Achieve Accurate Prognostic Classification and Identification of Therapeutic Targets in Stage III Colorectal Cancer
Source: Cancers (Basel). 2022 Apr 8;14(8):1895. doi: 10.3390/cancers14081895 (PMC9029745; doi:10.3390/cancers14081895)
Supplement: Supplementary file 1 [file cancers-14-01895-s001.zip › Supplementary Table S6. The clinical impact of radiomic features 20220327.pdf]

Supplementary Table S6. The clinical impact of adjusted radiomic features

Radiomic Feature	Measures	Recurrent Impact
wavelet.LHH_glcm_Idmn	Idm (inverse difference moment, a.k.a Homogeneity 2) measures the local homogeneity of an image. IDM weights are the inverse of the Contrast weights.	Positive; Idmn, local homogeneity, is associated with cancer recurrence.
wavelet.LHH_glcm_Idn	Idn (inverse difference normalized) is another measure of the local homogeneity of an image. Unlike Homogeneity1, IDN normalizes the difference between the neighboring intensity values by dividing the total number of discrete intensity values.	Positive; Idn, local homogeneity, is associated with cancer recurrence.
wavelet.LLH_glcm_Idn	Idn (inverse difference normalized) is another measure of the local homogeneity of an image. Unlike Homogeneity1, IDN normalizes the difference between the neighboring intensity values by dividing the total number of discrete intensity values.	Positive; Idn, local homogeneity, is associated with cancer recurrence.
wavelet.LHL_glcm_InverseVariance (IV)	In statistics, inverse-variance weighting is a method of aggregating two or more random variables to minimize the variance of the weighted average	Positive; IV is associated with cancer recurrence.
wavelet.HHH_gldm_DependenceVariance (DV)	DV (Dependence Variance): Measures the variance in dependence size in the image. Difference Variance measures heterogeneity that places higher weights on differing intensity level pairs that	Positive; DV, heterogeneity is associated with cancer recurrence.

	deviate more from the mean.	
wavelet.LHH_glszm_GrayLevelNonUniformityNormalized (GLNN)	GLNN (gray level non-uniformity normalized) measures the variability of gray-level intensity values in the image, with a lower value indicating a more significant similarity in intensity values. This is the normalized version of the GLN formula.	Negative; GLNN, similarity in intensity, is associated with cancer non-recurrence.
wavelet.LHH_gldm_LowGrayLevelEmphasis (LGLE)	LGLE (low gray level emphasis) measures the distribution of low gray-level values, with a higher value indicating a greater concentration of low gray-level values in the image.	Negative; LGLE is associated with cancer non-recurrence.
wavelet.LHH_glrlm_LowGrayLevelRunEmphasis (LGLRE)	LGLRE (low gray level run emphasis) measures the distribution of low gray-level values, with a higher value indicating a greater concentration of low gray-level values in the image.	Negative; LGLRE is associated with cancer non-recurrence.
wavelet.LHH_ngtdm_Contrast	Contrast is a measure of the spatial intensity change but depends on the overall gray level dynamic range. Contrast is high when both the dynamic range and the spatial change rate are high.	Negative; Contrast is associated with cancer non-recurrence.
wavelet.LHH_glszm_LowGrayLevelZoneEmphasis (LGLZE)	LGLZE (low gray level zone emphasis) measures the distribution of lower gray-level size zones. A higher value indicates a greater proportion of lower gray-level values and size zones in the image.	Negative; LGLZE, such as tumor necrosis or mucinous in CT scan image, is associated with cancer non-recurrence.
